# Comparing the defence-related gene expression changes upon root-knot nematode attack in susceptible *versus* resistant cultivars of rice

**DOI:** 10.1038/srep22846

**Published:** 2016-03-10

**Authors:** Chanchal Kumari, Tushar K. Dutta, Prakash Banakar, Uma Rao

**Affiliations:** 1 Division of Nematology, ICAR-Indian Agricultural Research Institute, New Delhi, 110012, India; 2 School of Biotechnology, Kalinga Institute of Industrial Technology, Bhubaneswar, 751024, India

## Abstract

Rice is one of the major staple food crops in the world and an excellent model system for studying monocotyledonous plants. Diseases caused by nematodes in rice are well documented and among them, root-knot nematode (RKN), *Meloidogyne graminicola*, causes extensive yield decline. It is therefore necessary to identify novel sources of natural resistance to RKN in rice and to investigate the rice-RKN interaction in detail to understand the basal plant defence mechanisms and nematode manipulation of the host physiology. To this end, six different cultivars of rice were initially screened for RKN infection and development; Pusa 1121 and Vandana were found to be most susceptible and resistant to RKN infection, respectively. In order to investigate the role of major hormone-regulated plant defence pathways in compatible/incompatible rice-RKN interaction, some well-identified marker genes involved in salicylate/jasmonate/ethylene pathway were evaluated for their differential expression through qRT-PCR. In general, our study shows a remarkable discrepancy in the expression pattern of those genes between compatible and incompatible rice-RKN interaction. As most information on the molecular interplay between plants and nematodes were generated on dicotyledonous plants, the current study will strengthen our basic understanding of plant-nematode interaction in the monocot crops, which will aid in defining future strategies for best plant health measures.

Plant-parasitic nematodes (PPNs) have proved to be one of the most difficult to manage and stubborn pest of agricultural crops. PPNs display a wide variety of interactions with their hosts. Most advanced of them are sedentary endoparasites, which induce a specialized feeding cell in the host tissue. These feeding structures are believed to serve as the singular nutrient source for the nematode development and reproduction. A plethora of nematode effector proteins have been identified which interact with the several plant proteins to initiate and maintain the feeding cell and usurps innate host defence^1^,^2^.

Being one of the major staple food crop, and a promising model monocotyledonous plant, rice (*Oryza sativa* L.) has garnered considerable attention from the nematologists studying the physiological and molecular interaction between rice and PPNs. Root-knot nematode (RKN), *Meloidogyne graminicola* is emerging as a serious bottleneck in rice-wheat cropping system of Indo-Gangetic plains and is causing substantial yield losses in all the rice growing belts of South-east Asia. The infective second-stage juvenile (J2) penetrates the rice root behind the root tip zone, traverses the vascular tissue and induces a typical feeding cell, known as the giant cell (GC). Cells surrounding the GC are hypertrophied to render the formation of macroscopic hook-like galls on the root system^3^,^4^,^5^.

To date, almost all grown *O. sativa* cultivars tested are known to be susceptible to RKN infection, while the non-cultivated African relatives, *O. glaberrima* and *O. longistaminata* are reported to be resistant to the infection of *M. graminicola*^3^,^5^,^6^,^7^,^8^. To meet the escalating demand for food owing to the growth of global population, improved rice varieties with superior yield potential and tolerance to biotic and abiotic stresses are being developed using molecular breeding approaches. Some of these cultivars include, Pusa 1121 (high-yielding basmati), Samba Mahsuri/BPT 5204 (diabetic friendly and resistant to bacterial leaf blight), Suraksha (resistant to gall midge), Vandana (drought tolerant, weed competitive, moderately resistant to sheath blight and blast disease), IC 81372 (accession, efficiently used for genetic transformation), Taipei 309 (model japonica rice for *in vitro* culture) etc. (Rice Knowledge Management Portal: www.rkmp.co.in)^9^,^10^. However, the nature of interaction of RKN with those cultivars has not yet been investigated. Hence, in order to fill the gaps in our understanding of host plant resistance to PPNs, a representative highly synchronous plant-PPN interaction model, such as rice-*M. graminicola* pathosystem may be dissected using both behavioural and molecular studies.

The small size of PPNs, as well as the fact that many species are obligate biotrophs and cannot be cultured in large numbers, makes them extremely difficult experimental organisms. Several assays have used agarose as a medium to study the nematode behaviour. However, nematodes migrate along the surface of the agar in two-dimensional way which is quite unlike of their movement in the soil environment^11^. The advent of Pluronic F-127 (PF-127) gel as the medium to study PPN behaviour, has revolutionized the concept of plant-nematode interaction at the initial level^12^,^13^,^14^,^15^. Nematodes suspended in PF-127 gel, can move freely in three dimensions in response to stable chemical gradients emanating from the host roots. This system mimics the natural soil environment that leads to the more realistic appraisal of host-pathogen interactions. Excellent transparency of the gel leads to better monitoring of PPN infection process around the host root tissues under the microscope. PF-127, a copolymer of propylene oxide and ethylene oxide, has negligible toxicity towards nematodes or plant tissues^12^,^16^. In the present study, we have assessed the suitability of an alternative, soil-free system (PF-127) for screening of six different varieties of rice, such as Pusa 1121, BPT 5204, Suraksha, Vandana, IC 81372 and Taipei 309, to identify the resistance/susceptibility response of those diverse germplasm to RKN infection.

Invasion of PPNs into a host plant triggers the constitutive and induced defence response in the infected plant which is regulated by the concerted expression of different plant signalling pathways, including plant hormones (ethylene, ET; salicylic acid, SA and jasmonic acid, JA), pathogenesis-related (PR) proteins, and various plant transcription factors; and outcome of this highly coordinated signalling responses ultimately determine the plant susceptibility/resistance to PPNs^17^,^18^,^19^. Nevertheless, understanding the role of phytohormones in inducing systemic and local defence in monocotyledons upon PPN infection is yet an underexploited territory. In this context, in the current study, the susceptible and resistant rice varieties identified in the PF-127 assay were challenge inoculated with *M. graminicola* and relative expression of several defence genes were studied in both the local and systemic plant tissues. Our study provides a global view of the defence-related gene expression changes in susceptible and resistant cultivars of rice upon nematode infection. Significant findings from this study may be extrapolated to understand the intimate molecular dialog governing the resistance/susceptibility in rice in response to RKN infection.

## Results

### Varietal screening of rice for nematode infection in PF-127 medium

**Figure 1:**
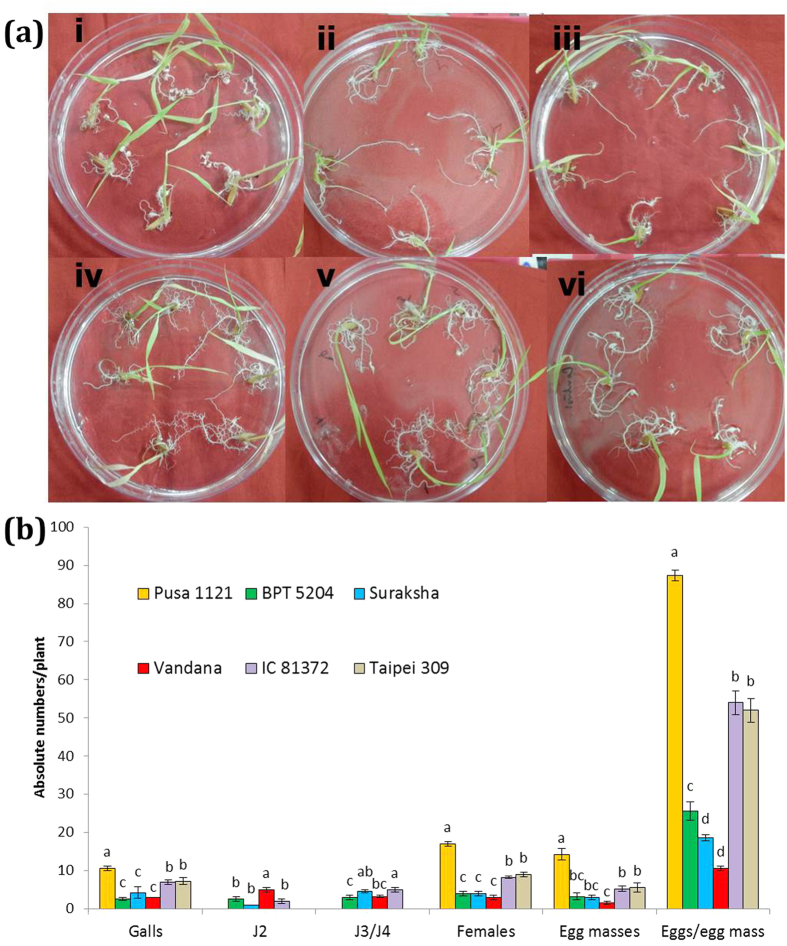
Screening of rice varieties for nematode infection. (**a**) Nematode infected plantlets of Pusa 1121 (i), BPT 5204 (ii), Suraksha (iii), Vandana (iv), IC 81372 (v) and Taipei 309 (vi) in the Petri dish containing PF-127 medium at 15 dpi. Typical hook-like galls were observed in all the varieties with greater number of them in Pusa 1121, IC 81372 and Taipei 309 compared to BPT 5204, Suraksha and Vandana. (**b**) Relative number of galls, J2, J3/J4, females, egg masses and eggs/egg mass of *M. graminicola* in different varieties of rice at 15 dpi. Different letters within any parameter are significantly different at *P* = 0.05 (J3: third-stage juveniles, J4: fourth-stage juveniles). Error bars indicate standard error of mean.

To assess the tolerance of six diverse rice varieties to RKN infection in a soil-free environment, root tips of each of the rice seedlings were inoculated with 30 J2s of *M. graminicola* in the Petri dish containing PF-127 medium and incubated for 15 days due to the short life cycle of RKN^3^,^4^. Among the different varieties, average number of galls per plant was found to be significantly higher (*P* < 0.05) in Pusa 1121, IC 81372 and Taipei 309 compared to BPT 5204, Suraksha and Vandana, with greatest number of them in Pusa 1121 at 15 dpi (days post inoculation) (Fig. 1a,b). Similar trend was observed for the number of females, egg masses and eggs/egg mass supported, while Pusa 1121 and Vandana harboured the greatest and least number of them, respectively (Fig. 1b). Even after 15 dpi, intermediate nematode development stages, such as J2s and J3/J4s were observed in BPT 5204, Suraksha, Vandana and IC 81372, indicating the sluggish development of *M. graminicola* in those varieties (Fig. 1b). Multiplication factor (MF) which reflects the overall ability of the nematode to be a successful parasite varied significantly among all the varieties. Calculated MF value of *M. graminicola* was found to be greatest in Pusa 1121 (41.65) followed by IC 81372 (9.68) and Taipei 309 (10.06) and least in BPT 5204 (2.98), Suraksha (1.9) and Vandana (0.57). Based on all the parameters, Pusa 1121 (susceptible to RKN) and Vandana (resistant to RKN) were selected for further studies.

### Comparative invasion, development and reproduction of *M. graminicola* in the susceptible *versus* resistant cultivar of rice

**Figure 2:**
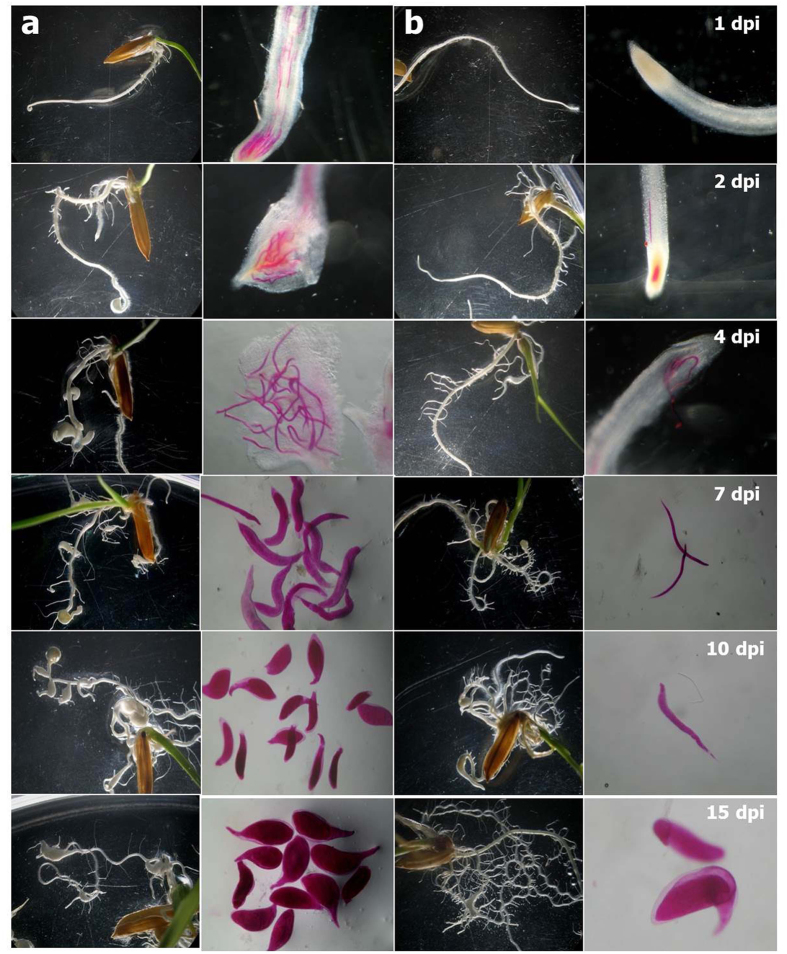
Comparative life cycle progression of *Meloidogyne graminicola* in susceptible and resistant varieties of rice. (**a**) - Pusa 1121, (**b**) - Vandana. Formation of gall was evident at 2 dpi in Pusa 1121. Pusa 1121 had supported more number of nematodes with higher galling intensity throughout the development process compared to Vandana. At 10 dpi, J2s were developed to young females in Pusa 1121 and J3/J4s in Vandana, suggesting that the nematode development was delayed in Vandana compared to Pusa 1121. Nematodes were stained with acid fuchsin.

Significantly more J2s (*P* < 0.05) had penetrated the roots of Pusa 1121 compared to Vandana at 1, 2, 3 and 4 dpi. Accordingly, J2s were developed to J3 and J4 stages in significantly greater number (*P* < 0.05) in Pusa 1121 than Vandana at 5 and 7 dpi. Faster development and reproduction was recorded in Pusa 1121 in which nematodes attained the young female stage at 10 dpi, adult females at 12 and 15 dpi, and started producing eggs at 15 dpi in significantly greater numbers (*P* < 0.05). On the contrary, delayed penetration, development and reproduction were observed in Vandana (Table 1, Fig. 2). Calculated MF value of *M. graminicola* was found to be greatest in Pusa 1121 and least in Vandana (Table 1).

Root swellings resulting from galls, initially formed at the root tip of Pusa 1121 were visible at 2 dpi. Subsequently, the infected roots stopped their growth and galls were formed at the lateral roots due to the progression of nematode damage. At 10 and 15 dpi, increased number of lateral root emergence was observed in Vandana compared to Pusa 1121, indicating the formation of structural barrier in roots to prevent the subsequent invasion by nematodes (Fig. 2).

### Innate defence response in root and shoot tissues of rice upon RKN infection

In order to gain an insight into the differential response of susceptible and resistant rice cultivars upon RKN infection, expression of genes involved in plant innate immunity was investigated in infected root and systemic shoot tissues at 2 and 6 dpi using quantitative real-time PCR (qRT-PCR).

**Figure 3:**
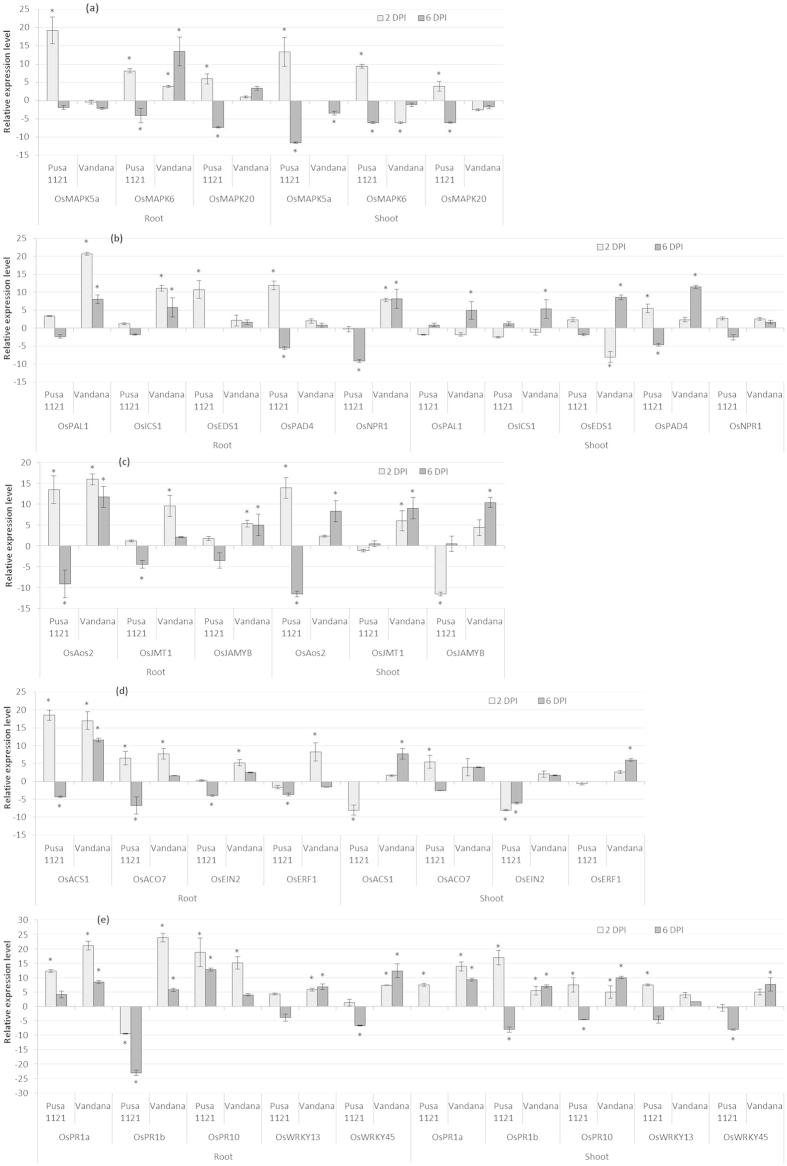
Differential expression patterns of defence-related genes in the root and shoot tissues of susceptible and resistant cultivars of rice infected with *Meloidogyne graminicola*. (**a**) MAPK-related response, (**b**) SA-related response, (**c**) JA-related response, (**d**) ET-related response, and (**e**) General defence response. Gene expression was measured by qRT-PCR in plants infected with *M. graminicola* at 2 and 6 dpi. Gene expression levels were normalized using two internal reference genes, *Os18srRNA* and *Os-actin*. Data are shown as the log_2_-transformed values of the fold change levels of infected root and shoot in comparison with the control tissue (i.e. root and shoot of uninfected plants). Bars represent mean expression levels and SE from two biological and three technical replicates each containing a pool of ten plants. Asterisks indicate significant differential expression (*P* < 0.05) in comparison with uninfected plants.

*OsMAPK5a*, *OsMAPK6* and *OsMAPK20*, encode mitogen-activated protein kinases (MAPK) which are involved as phosphorylation cascades in both pathogen associated molecular pattern (PAMP)-triggered immunity (PTI) and effector-triggered immunity (ETI) during early response to pathogens. Along with that, these proteins play important role in plant signal transduction related to biotic stresses^20^,^21^,^22^. Early upon infection (2 dpi), the mRNA levels of *OsMAPK5a*, *OsMAPK6* and *OsMAPK20* were considerably upregulated followed by the downregulation at 6 dpi in the infected roots *vis-à-vis* shoot tissues of Pusa 1121 (Fig. 3a). On the other hand, although *OsMAPK6* and *OsMAPK20* levels were overexpressed in the infected root of Vandana, transcription of *OsMAPK5a* was attenuated at both at 2 and 6 dpi. Surprisingly, *MAPK* genes were either downregulated or returned to basal expression level in the infected shoots of Vandana in comparison to uninfected control (Fig. 3a).

Overall, it seems that although the MAPK-mediated defence mechanism is activated early at 2 dpi, the nematodes were able to suppress that by 6 dpi in susceptible plants. Conversely, a non-significant expression of *OsMAPK5a* and *OsMAPK20* was documented in the RKN-infected root of Vandana at both time points, suggesting the equivocal role of *MAPK* genes in induced systemic defence of rice against RKNs. Additionally, MAPK-mediated defence signal was not translocated to the shoot tissues even after 6 dpi in Vandana, thereby preventing plants to warn their systemic tissues of RKN presence.

### SA-dependent responses in root and shoot tissues of rice upon RKN infection

*OsPAL1*, *OsICS1* (key enzymes in the salicylate biosynthetic pathway^23^), *OsEDS1*, *OsPAD4* (modulates SA-upstream signalling^24^,^25^) and *OsNPR1* (interacts with TGA transcription factors and a key mediator of systemic acquired resistance or SAR^26^,^27^) were used as the marker genes for studying SA-related responses.

Local endogenous levels of *OsPAL1* and *OsICS1* were slightly induced at 2 dpi followed by the weak downregulation at 6 dpi in the roots of Pusa 1121 in response to RKN infection. Vice versa was observed in the shoot tissues of Pusa 1121. On the contrary, mRNA levels of *OsPAL1* and *OsICS1* were significantly and consistently elevated in the infected root of Vandana at both 2 and 6 dpi. However, in the shoots of Vandana, transcripts of *OsPAL1* and *OsICS1* were strongly expressed only at 6 dpi (Fig. 3b). The steady-state mRNA levels of *OsEDS1* and *OsPAD4* were either returned to non-infected tissue levels or significantly repressed at 6 dpi before being sigficantly increased at 2 dpi in the RKN-infected roots of Pusa 1121. Similar response was observed in the shoot tissues of Pusa 1121. By contrast, although slightly upregulated, temporal expression of *OsEDS1* and *OsPAD4* was not significantly altered in *M. graminicola*-infected root of Vandana compared to corresponding mock-inoculated tissue. Interestingly, in the shoot tissues of Vandana, *OsEDS1* and *OsPAD4* levels were significantly upregulated at 6 dpi (Fig. 3b). Expression of *OsNPR1* was not differentially expressed or downregulated in Pusa 1121 roots in contrast to consistent and significant upregulation in Vandana at both time points. Although, expression pattern of *OsNPR1* was not significantly different in shoot tissues of either cultivar (Fig. 3b).

To this end it is assumed that, EDS1- and PAD4- mediated SA-upstream signalling, which had triggered the induced defence against RKN at the early stage of infection, was negatively regulated during the later stage of infection in susceptible plants. On the other hand, the resistant cultivar, such as Vandana had shown substantial upregulation of SA biosynthesis and responsive (NPR1-mediated) genes in the infected roots during both early and late infection of RKN. However, the same could not be documented in the infected roots of Vandana for SA signalling (EDS1- and PAD4- mediated) genes. In Vandana, SA biosynthesis and signalling was inhibited during the early stage of plant-nematode interaction just before the plants were able to send out warning signals to systemic tissues at 6 dpi. Therefore, considering the ambivalent expression of SA-related genes in the resistant and susceptible cultivars, it can be speculated that the role of SA in inducing systemic defence in rice upon RKN infection is less direct than that of other defence regulatory phytohormones.

### JA-dependent responses in root and shoot tissues of rice upon RKN infection

*OsAOS2* (a key enzyme in JA biosynthesis^28^), *OsJMT1* (converts JA to volatile MeJA^29^) and *OsJAMYB* (JA-inducible Myb transcription factor^30^) were used as the marker genes to investigate the JA-related responses.

Early upon infection, in the roots of both Vandana and Pusa 1121, a significantly enhanced transcript accumulation of *OsAOS2* was recorded, which continued to be upregulated in Vandana but rather showed significant repression in Pusa 1121 at 6 dpi. Corroborating with this observation, *OsJMT1* and *OsJAMYB* was differentially expressed in the roots of Pusa 1121 and Vandana in a pattern similar to *OsAOS2* except the fact that *OsJMT1* was weakly upregulated in Vandana at 6 dpi (Fig. 3c). An identical response of *OsAOS2* was recorded in the shoot tissues of different rice cultivars. Concomitantly, almost no transcriptional alteration or negative regulation of *OsJMT1* and *OsJAMYB* was detected in the shoots of Pusa 1121 compared to constitutive expression of those genes in Vandana (Fig. 3c). Taken together, these data indicate that unlike Pusa 1121, JA plays major role during the early and late defence response of Vandana to RKN infection.

### ET-dependent responses in root and shoot tissues of rice upon RKN infection

*OsACS1*, *OsACO7* (two major catalytic enzymes involved in the biosynthesis of ET from methionine^31^), *OsEIN2* (central signal transducer in ET signalling pathway^32^) and *OsERF1* (ET-inducible gene^33^) were used as the marker genes to demonstrate the ET-related responses.

Despite upregulated at 2 dpi mRNA levels of *OsACS1* and *OsACO7* were attenuated in the infected root of Pusa 1121 at 6 dpi. A near baseline expression or downregulation of *OsEIN2* and *OsERF1* was documented in the infected root of Pusa 1121 at both 2 and 6 dpi. On the contrary, a strong induction of *OsACS1* was recorded in the roots of Vandana at both 2 and 6 dpi. However, the transcripts of *OsACO7*, *OsEIN2* and *OsERF1* were not significantly induced at 6 dpi before being highly expressed at 2 dpi in Vandana (Fig. 3d). ET biosynthesis and signalling genes were either downregulated or not differentially expressed in the shoot of the susceptible plant at both time points. By contrast, although a minor upregulation was observed in few instances, systemic mRNA levels of *OsACS1*, *OsACO7*, *OsEIN2* and *OsERF1* were consistently upregulated in the shoots of Vandana at both 2 and 6 dpi (Fig. 3d). Collectively, a consistent overexpression of ET responsive genes throughout the course of nematode infection in the resistant plants suggests a positive correlation between ET-inducible gene expression in rice and overall defence to *M. graminicola*.

### General defence responses in root and shoot tissues of rice upon RKN infection

To further elucidate the molecular machinery underpinning the general defence response of rice triggered upon nematode infection, the differential expression of three PR genes (*OsPR1a*, *OsPR1b*, *OsPR10*)^34^ was assessed. Additionally, the expression of *OsWRKY13* and *OsWRKY45*, which acts as the positive transcriptional regulator of defence genes^22^,^35^,^36^, was also evaluated.

The strong upregulation of *OsPR10* and *OsPR1a* in all the varieties at 2 and 6 dpi confirms the defence-inducing capabilities of PR genes in rice roots in response to RKN attack. However, the strongest upregulation of *OsPR1a* and *OsPR1b* was recorded in Vandana at 2 dpi. Conversely, expression of *OsPR1b* was markedly downregulated at both 2 and 6 dpi in the infected root of Pusa 1121 (Fig. 3e). Increased and consistent transcript abundance of *OsPR1a*, *OsPR1b* and *OsPR10* was recorded in the shoots of resistant cultivar at 2 and 6 dpi. While in shoots of Pusa 1121, transcripts of *OsPR1a*, *OsPR1b* and *OsPR10* were either unaltered or attenuated at 6 dpi before being significantly expressed at 2 dpi (Fig. 3e). Despite being root-inducible, systemic mRNA levels of *OsWRKY13* did not significantly change upon RKN infection in the shoots of Vandana at any time point. Combining both the root and shoot expression data, transcripts of *OsWRKY45* were significantly upregulated at 6 dpi in Vandana in contrast to non-significant expression or downregulation in Pusa 1121 at any time point (Fig. 3e). In concordance with these findings, it appears that *OsWRKY13* has a minor positive effect in activating the systemic defence of rice in response to RKN attack.

### Expression of lignin and callose-related genes in rice roots during early attack of RKN

Lignin and callose deposition in plant roots reinforce the plant resistance to invading pathogens including PPNs^37^,^38^ by conferring mechanical strength to plant secondary cell walls. As revealed by infection bioassay (Table 1, Fig. 2) in the present study, *M. graminicola* J2s were able to invade the roots of Vandana in lesser number compared to Pusa 1121, during early stage of infection. This finding is attributable to the possible role of lignin and callose-related genes in rice basal defence against nematodes. With this speculation, the expression pattern of two lignin biosynthesis genes (*OsC4H*, *OsCAD6*)^39^, three callose synthase genes (*OsGSL1*, *OsGSL3*, *OsGSL5*)^40^ and one callose hydrolysing gene (*OsGNS5*)^40^ in the RKN-infected root of Pusa 1121 and Vandana was investigated by qRT-PCR. Additionally, the expression data of *OsPAL1* (as an essential enzyme in the phenylpropanoid pathway catalyses the deamination of phenylalanine to transcinnamic acid, a precursor in lignin biosynthetic pathway)^41^ was also taken into account.

**Figure 4:**
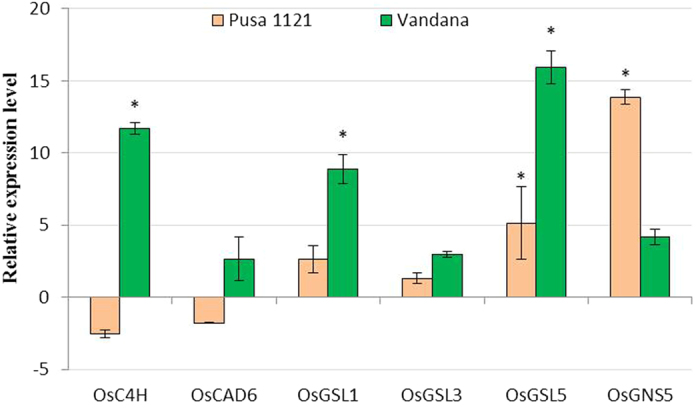
Differential expression patterns of genes involved in lignin biosynthesis and callose deposition in the root tissues of susceptible and resistant cultivars of rice infected with *Meloidogyne graminicola*. Gene expression was measured by qRT-PCR in plants infected with *M. graminicola* at 2 dpi. Gene expression levels were normalized using two internal reference genes, *Os18srRNA* and *Os-actin*. Data are shown as the log_2_-transformed values of the fold change levels of infected tissue in comparison with the control tissue (root tips of uninfected plants). Bars represent mean expression levels and SE from two biological and three technical replicates each containing a pool of ten plants. Asterisks indicate significant differential expression (*P* < 0.05) in comparison with uninfected plants.

At 2 dpi, *OsC4H* and *OsPAL1* was significantly upregulated in RKN-infected root of Vandana compared to uninoculated root tips. On the contrary, a slight but insignificant inhibition of *OsC4H* and a minor upregulation of *OsPAL1* occurred in the RKN-treated root of Pusa 1121 (Figs 3b and 4). However, expression of *OsCAD6* did not respond significantly to RKN infection at 2 dpi (Fig. 4). Nematode invasion in both susceptible and resistant cultivars produced the overexpression of *OsGSL1*, *OsGSL3* and *OsGSL5* at 2 dpi, although the callose synthase genes were expressed in comparatively greater amount in the roots of resistant plants. In agreement with these results, quantitatively greatest expression of *OsGNS5* was recorded in the RKN-infected root of susceptible plants compared to resistant ones at 2 dpi (Fig. 4). These findings support the notion that genes involved in lignin and callose deposition may play the pivotal role in inhibiting nematode penetration and consequently, the delayed development and reproduction of PPN occurs in resistant cultivar.

## Discussion

A simple, novel method for *in vitro* inoculation of plant roots with infective J2 is more advantageous for continuous nematode development studies compared to labour-intensive pot-based experiments. In accordance with this presumption, here, we demonstrate that PF-127 is arguably the appropriate matrix for monitoring the progression of nematode development inside plant roots for long periods of time in non-axenic environment, barring the complications related to lengthy sterilization process of nematodes for agar plate experiments. Even after successful sterilization, nematodes may get trapped in water films formed in the agar plate^13^. On the other hand, non-rigid, uniform texture and transparent nature of PF-127 unequivocally allows the nematodes to move freely in three-dimension inside the matrix. Earlier, entire development of potato cyst nematode, *Globodera pallida* in the solanaceous plants (*Solanum tuberosum* and *S. sisymbriifolium*) was investigated using PF-127 medium^42^. Information generated from the present study can be potentially utilized for screening resistant or transgenic crop varieties for nematode infection, multiplication, development and reproduction. Additionally, various nematode parasitic stages can be isolated from plant tissues with greater ease for genomic, proteomic or transcriptomic analyses.

Based on the outcome of screening bioassay, Pusa 1121 was found to be the most susceptible and Vandana was the most resistant to *M. graminicola* infection at 15 dpi. Subsequently, these varieties were subjected to nematode development studies for deeper understanding of nematode disease progression in susceptible *versus* resistant cultivars in real-time. Results suggest that *M. graminicola* had penetrated, developed and reproduced faster as well as in greater number in Pusa 1121 compared to Vandana. Previously, Vandana was showed to be moderately resistant to the field population of *M. graminicola* in pot culture conditions^43^. Nevertheless, factors determining the genotype-dependent resistance mechanism of rice in response to RKN infection remain to be determined.

A repertoire of genes are likely to be involved in stress and defence responses, signal transduction and phytohormone regulation in plant tissues in response to nematode infection^44^,^45^,^46^,^47^,^48^. In agreement with those reports, genes induced in those categories were differentially expressed in the present investigation. However, while comparing the response of susceptible *versus* resistant varieties upon infection with RKN, many discrepancies arose may be due to nematode effects (nematode biology varies with resistant and susceptible hosts), systemic hormone signalling effects or tissue-specific differential expression of selected genes. As a general trend, some remarkable differences and similarities were observed which are being discussed in the following paragraphs.

MAPK cascades are evolutionarily conserved, intracellular signalling modules that play decisive role in host responses to multiple biotic stresses by activating the PTI, ETI and PR gene induction^20^,^22^. Considering the redundancy of experimental reports in interaction of novel dicot plants such as Arabidopsis and tobacco with disease causing pathogens^20^,^49^, our understanding of the role of MAPK cascades in inducing innate defence of monocotyledons in response to PPNs are rather limited. In line with this view, we examined that whether the overexpression or suppression of *MAPK* genes in rice is an important determinant in conferring resistance or susceptibility to *M. graminicola*. An early transient activation of *OsMAPK5a*, *OsMAPK6* and *OsMAPK20* followed by their suppression at later stage in susceptible plants may relate to the capitulation to stress resulting from the development of root-knot disease. In resistant plants, transcription of *OsMAPK5a* was either unaltered or downregulated, which is in line with the fact that *OsMAPK5* negatively modulates PR gene expression in rice^20^,^29^. Knock-down of *OsMAPK5* in rice led to the enhanced resistance to fungal and bacterial pathogens^20^. In systemic shoots of Vandana, *OsMAPK6* and *OsMAPK20* was apparently downregulated, which exemplifies the equivocal role of MAPK cascades in conferring RKN resistance to Vandana.

Downstream of PTI and ETI activation, a coordinated expression of SA/JA/ET-mediated signalling renders the plant to effectively tailor its local and systemic defence against pathogens^19^,^50^. In addition, a SAR response is generated in infected tissues which is characterised by the local and systemic augmentation of endogenously synthesized SA and expression of PR proteins^25^. In dicot plants, SA pathway plays major role in eliciting defence against biotrophs that exclusively feed and reproduce on living host cells, in contrast to involvement of JA/ET pathways in defence against necrotrophs that kills the host cells during infection^51^. However, the role of phytohormones in inducing systemic and local defence in monocotyledons remains to be further deciphered. Our data show that SA production through *OsPAL1* and *OsICS1* is significantly upregulated in local (2 and 6 dpi) and systemic (6 dpi) tissues of Vandana compared to non-significant alteration in Pusa 1121, suggesting the significant role of SA biosynthetic pathway in protecting the resistant plants during nematode invasion.

As the key mediators of antagonistic cross-talk between SA and JA pathway, *EDS1* and *PAD4* are involved in upstream of SA production and signalling for basal resistance to biotrophs and hemi-biotrophs^19^. Our results show that RKN can actively suppress the *OsEDS1* and *OsPAD4* levels in the root of susceptible cultivar at later stage of infection compared to insignificant induction of those genes in resistant cultivar. Downstream SA signalling via *OsNPR1* is reported to be crucial for defence against rice bacterial blight and fungal blast^26^,^27^. In our study, local endogenous levels of *OsNPR1* were constitutively expressed in the resistant varieties in contrast to repression in susceptible one. However, contradicting expression data of *OsEDS1*, *OsPAD4* and *OsNPR1* in the shoot tissues of resistant plants indicate that perhaps transduction of SA signalling and responsive genes were impaired in the systemic tissues of rice during early incompatible interaction with RKN. Despite the fact that SA biosynthesis and signalling are positively correlated to SAR in other plants^52^, the exact role of SA in inducing SAR in rice still remains elusive^5^,^44^,^46^,^47^. In light of the outcome of present study, we hypothesize that SA signalling and responsive genes may play negligible role in innate immunity of rice against RKN.

The paramount importance of JA in inducing SAR, rather than SA, has been proposed in rice by several researchers^53^,^54^,^55^. Remarkably, our experiments revealed the consistent local and systemic induction of JA biosynthesis and responsive genes both at early and late stage of RKN infection in Vandana in comparison to variable response of those genes in Pusa 1121. It is known that the ET pathway modulates the strong activation of JA biosynthesis and signalling genes to induce the systemic defence in rice against *M. graminicola*^44^. This apprehension prompted us to assess the ET-induced defence in compatible and incompatible interaction of rice and *M. graminicola*. Mostly, a significantly enhanced transcript accumulation of ET biosynthesis, signalling and responsive genes were detected in the local and systemic tissues of resistant cultivars compared to RKN-induced suppression of those genes in susceptible plants. Taken together, our data reinforces the knowledge that JA pathway, presumably modulated by ET, can efficiently contribute to resistance response of rice to RKN invasion.

Mounting evidence indicates that WRKY transcription factors constitutively regulate the expression of defence-related genes and their differential expression is attributable to the resistant or susceptible reaction of plants to nematodes^19^. Except insignificant expression in shoot tissues, *OsWRKY13* and *OsWRKY45* was overexpressed in the early and late RKN-infected root of Vandana in our study. However, in susceptible root and shoot, *OsWRKY13* and *OsWRKY45* were upregulated early but later downregulated.

Given the ultimate importance of PR genes in eliciting SAR, our results demonstrate that *OsPR1a* and *OsPR10* were consistently upregulated in the roots of both susceptible and resistant cultivars during early and late RKN infection. By contrast, transcription of *OsPR1b* was significantly attenuated in the susceptible root at both time points. Regarding shoot tissues, unlike the resistant cultivars expression of all PR genes were either downregulated or returned to basal level in susceptible cultivar at later stage of infection. According to earlier reports, several PR genes were suppressed in the systemic shoot tissues of Arabidopsis and tomato during their compatible interaction with RKN^56^,^57^. Likewise, the most evident difference in PR gene accumulation between the compatible and incompatible interaction of tomato and *M. incognita* was recorded in the shoot tissues^58^.

Accumulation of lignin and callose strengthens the plant cell wall to limit the accessibility of cell wall-degrading enzymes secreted by PPNs during penetration and migration in plant roots^59^. In corroboration with this view, during early interaction of rice and *M. graminicola*, comparatively greater induction of lignin and callose biosynthesis genes in the roots of resistant cultivars than the susceptible ones were recorded in the current study. In parallel, a callose-degrading gene was quantitatively greatly induced in the susceptible cultivar compared to the resistant ones. Therefore, lignin and callose-mediated plant basal defence may inhibit the penetration and delay the development of RKNs in the root of resistant varieties.

**Figure 5:**
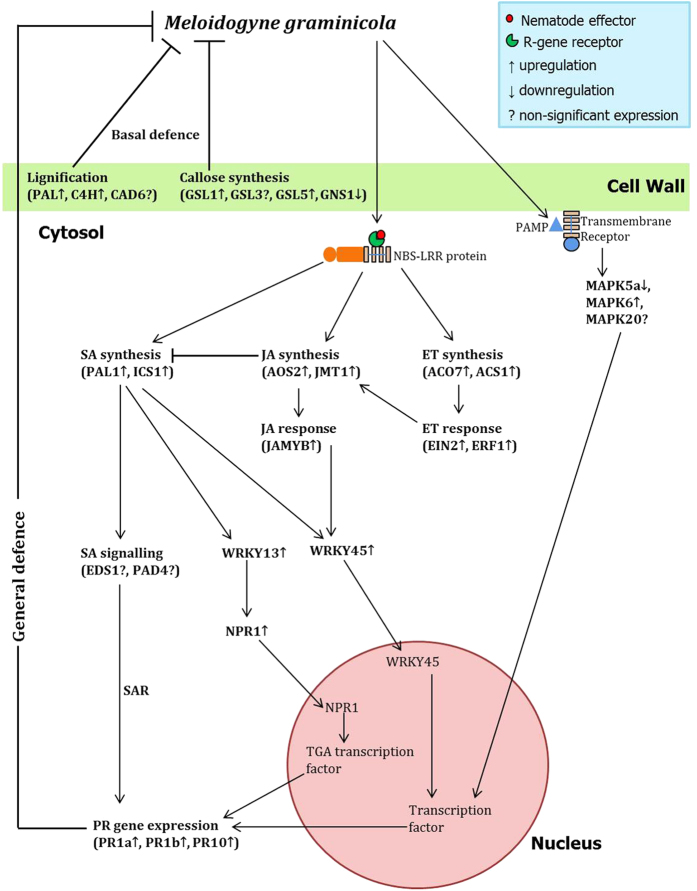
Model depicting the coordinated signalling network in rice leads to induction of local and systemic defence against *Meloidogyne graminicola* invasion. Formation of structural barrier to nematode penetration is attributed to lignin and callose deposition in plant cell walls, whereas systemic hormone-mediated defence is initiated upon recognition of nematode secreted effectors and PAMPs by plant transmembrane receptors and R-proteins. Genes involved in different pathways are indicated in parentheses. Lines ending with arrows indicate activation. Lines ending with a perpendicular short line indicate suppression or antagonistic interaction.

Considering the inadequately represented perspective of the role of plant innate immunity in incompatible plant-PPN interaction, our study demonstrates the trend in concerted differential expression of a battery of defence-related genes in compatible *versus* incompatible rice-RKN interaction, which can be translated to elucidate the molecular logic in determining factors governing resistance/susceptibility of plants to nematodes. Our data demonstrates that early upon infection, basal host defences are activated in both susceptible and resistant plants, whereas it is ostensibly suppressed during later stage of infection in susceptible plants. Specifically, genes involved in SA biosynthesis (but not SA signalling), JA and ET pathway and PR genes have positive effect on resistance response of rice to nematode infection. On the other hand, during compatible interaction RKN interfere with the hormone homeostasis of plants to suppress the systemic defence signalling, and, consequently, progression of nematode disease occurs due to establishment and maintenance of functional feeding site in susceptible plants. As illustrated by shoot expression data, transfer of root-synthesized defence genes from infected resistant roots to shoots largely at later stage of infection, indicates the successful induction of SAR in resistant cultivars. When considered together, our results support a model (Fig. 5) in which successful induction of both local and systemic defence genes leads to incompatible rice-*M. graminicola* interaction.

Further investigation is necessary to gain an insight into the role of nematode effectors in suppressing or expressing the innate defence of rice in compatible and incompatible interaction. Chorismate mutase, secreted via the stylet of *Heterodera glycines* was suggested to be the possible virulence factor for cyst nematode infection in soybean^60^. An effector of *H. schachtii*, 10A06, interacts with Arabidopsis spermidine synthase to suppress the SA responsive genes in plants^61^. Root-knot nematode calreticulin, Mi-CRT is a key effector in defence suppression of Arabidopsis^62^. Apart from that, continued investigation on cross-talks of different signalling molecules will unveil another dimension of hormone-induced defence pathways in rice.

## Methods

### Culturing of nematodes

A pure culture of an Indian isolate of *M. graminicola* Golden & Birchfield was maintained on rice (*O. sativa* L. cv. Pusa 1121) in a glasshouse. Egg masses were collected from the galled roots of infected plant using sterilized forceps and were kept for hatching in a double-layered paper tissue supported on a moulded sieve of wire gauze in a Petri dish containing distilled water. Freshly hatched J2s were used for all the experiments.

### Germination of seeds

Seeds of different cultivars of rice, i.e. Pusa 1121, BPT 5204, Suraksha, Vandana, IC 81372 and Taipei 309 (kindly provided by the Division of Genetics and Division of Plant Physiology, ICAR-Indian Agricultural Research Institute, New Delhi) were soaked overnight in distilled water and surface-sterilized with 70% ethanol for 30 s followed by three times rinsing in sterile water. Sterilized seeds were placed in wet filter paper inside the Petri dish and incubated in a growth chamber at 28 °C. Seedlings of 3–4 days old were used for the following experiments.

### Screening of varieties for nematode infection in Pluronic gel medium

Pluronic F-127 (PF-127) (Sigma-Aldrich) gel was prepared as previously described^12^,^14^,^15^. Each of the rice varieties listed above was screened for RKN infectivity in the standard Petri dishes (110 × 25 mm, HIMEDIA). Twenty five ml of 23% PF-127 were poured into each Petri dish containing seven, uniformly distributed seedlings of the identical variety at 15 °C. Approximately 30 J2s of *M. graminicola* were inoculated at the root tip of each seedling using a pipette tip followed by setting of gel at the room temperature. The covered Petri plates were placed in a humid tray and incubated at 28 °C with 16:8 h light:dark photoperiod in a growth chamber. Three plates for each variety were included in each experiment and each experiment was repeated at least twice.

At 15 dpi, plantlets were harvested from the gel by simply placing the Petri dishes briefly over an ice bath. Due to the slight decrease in temperature, gel was liquefied and allowed the plantlets to be easily extracted sans any damage to the root system. Roots were stained with acid fuchsin^63^ and the number and stage of the invaded nematodes were observed after dissecting the stained galls under the microscope. To determine the reproductive potential of *M. graminicola* in different varieties, nematode multiplication factor [(number of eggmasses × number of eggs per egg mass) ÷ nematode inoculum level] was calculated. Photographs were taken in a Zeiss Axiocam MRm microscope.

### Nematode development assays in Pluronic gel medium

Based on the outcome of varietal screening assay, two of the rice varieties, such as Pusa 1121 and Vandana were used to study the comparative development of *M. graminicola* in PF-127 medium. Root tip of the germinated seedlings was infected with the RKN and Petri plates were incubated as described above. Three plates for each variety were included in each experiment and each experiment was repeated at least twice. The plantlets were removed from the medium daily starting from the 1 dpi up to 15 dpi. Roots were stained and dissected under the microscope to identify the different developmental stages of RKN. Photographs were taken in a Zeiss Axiocam MRm microscope.

### Expression analysis of rice defence genes in response to RKN infection

Root tip of the rice cultivars – Pusa 1121 and Vandana – were inoculated with the J2s of *M. graminicola* in PF-127 medium as documented above. At 2 and 6 dpi, plantlets were harvested from the medium, excised root tips and shoots were immediately and separately frozen in liquid nitrogen and stored at –80 °C until use.

Total RNA was isolated from the root tip and shoot of infected and non-infected rice seedlings using NucleoSpin total RNA Kit (Macherey-Nagel, Germany), with addition of an on-column DNase I digestion. Extracted RNA was assessed for quality and quantity using Nanodrop ND-1000 spectrophotometer (Thermo Scientific). Approximately 500 ng of the purified RNA was reverse transcribed to cDNA using cDNA synthesis Kit (Superscript VILO, Invitrogen). Further, cDNA was used for amplification of few candidate defence genes of rice, such as *MAPK5a, MAPK6, MAPK20* (plant innate immunity), *PAL1*, *ICS1* (SA biosynthesis), *EDS1*, *PAD4* (SA signalling), *AOS2* (JA biosynthesis)*, JMT1* (MeJA biosynthesis), *JAMYB* (JA response), *ACO7*, *ACS1* (ET biosynthesis), *EIN2* (ET signalling), *ERF1* (ET response), *NPR1*, *WRKY13*, *WRKY45* (transcription factors), *PR1a*, *PR1b*, *PR10* (SAR marker genes), and *C4H*, *CAD6*, *GSL1*, *GSL3*, *GSL5*, *GNS5* (induced structural defence). Primer details are given in the Supplementary Table S1.

To analyse the transcript abundance of above-mentioned genes, qRT-PCR was carried out in a realplex^2^ thermal cycler (Eppendorf) using SYBR Green Supermix Kit (Eurogentec). Reaction mixture for each sample contained a final volume of 10 μl, comprising of 5 μl of SYBR Green PCR Master mix (Eurogentec), 750 nM of each primer and 1.5 ng of cDNA. The cycling conditions were as follows: a hot start of 95 °C for 5 min, followed by 40 cycles of 95 °C for 15 s and 60 °C for 1 min. The specificity of amplification was determined by an additional melt curve programme (95 °C for 15 s, 60 °C for 15 s, followed by a slow ramp from 60 °C to 95 °C). Two constitutively expressed genes, *Os18SrRNA* and *Os-actin*, were used for normalization of qRT-PCR data (Supplementary Table S1). At least two biological and three technical replicates were used for each of the samples. In order to determine the relative gene expression in different rice cultivars, mean Ct values were obtained and fold change values were calculated using 2^−ΔΔCT^ method^64^. The non-infected rice was treated as the control. The results are expressed as the log2-transformed fold change values.

### Statistical analysis

Data of the bioassay experiments were subjected to one way Analysis of variance. Results are reported as significant or non-significant based on Duncan’s multiple comparison test with significance level at *P* < 0.05 using SAS software (version 9.3). Regarding qRT-PCR data, significant differential expression between infected plants and control tissue was determined by student’s *t*-test at *P* < 0.05.

## Additional Information

**How to cite this article**: Kumari, C. *et al.* Comparing the defence-related gene expression changes upon root-knot nematode attack in susceptible *versus* resistant cultivars of rice. *Sci. Rep.* **6**, 22846; doi: 10.1038/srep22846 (2016).

## Supplementary Material

Supplementary Information

## Figures and Tables

**Table 1:** Comparison of *Meloidogyne graminicola* invasion, development and reproduction in susceptible and resistant cultivars of rice.

Life cycle stages	Days post inoculation	Number of nematodes/root system	
		Pusa 1121	Vandana
J2	1	22 ± 1.53^b^	0
	2	25.66 ± 1.20^a^	1.33 ± 0.33^e^
	3	22.66 ± 1.76^b^	2.33 ± 0.33^e^
	4	27 ± 1.15^a^	3.33 ± 0.33^de^
	5	5.33 ± 0.88^d^	3.33 ± 0.33^de^
	7	1.33 ± 0.33^e^	3.66 ± 0.33^de^
J3/J4	5	18 ± 1.15^b^	1.33 ± 0.33^c^
	7	22.66 ± 1.45^a^	1.66 ± 0.33^c^
	10	2.33 ± 0.33^e^	1.33 ± 0.33^c^
	12	0	2 ± 0.58^c^
Females	10	23.33 ± 0.88^b^	0
	12	24 ± 0.58^ab^	1.33 ± 0.67^c^
	15	26 ± 0.58^a^	2.66 ± 0.33^c^
Egg masses	15	23 ± 1.53^a^	2.66 ± 0.33^b^
Eggs/egg mass	15	91.66 ± 1.67^a^	10 ± 0.58^b^
MF value		70.33 ± 5.17^a^	0.89 ± 0.13^b^

Means ± standard errors are presented. Different letters within any parameter (J2, J3/J4, Females, Egg masses, Eggs/egg mass and MF) are significantly different at *P* = 0.05.

## References

[b1] Gheysen G. & Mitchum M. G. How nematodes manipulate plant development pathways for infection. Curr. Opin. Plant. Biol. 14, 415–421 (2011). 21458361 10.1016/j.pbi.2011.03.012

[b2] Jones J. T. *et al.* Top 10 plant-parasitic nematodes in molecular plant pathology. Mol. Plant Pathol. 14, 946–961 (2013). 23809086 10.1111/mpp.12057 PMC6638764

[b3] De Waele D. & Elsen A. Challenges in tropical plant nematology. Ann. Rev. Phytopathol. 45, 457–485 (2007). 17489690 10.1146/annurev.phyto.45.062806.094438

[b4] Dutta T. K., Ganguly A. K. & Gaur H. S. Global status of rice root-knot nematode, *Meloidogyne graminicola. Afr*. J. Microbiol. Res. 6, 6016–6021 (2012).

[b5] Kyndt T., Fernandez D. & Gheysen G. Plant-parasitic nematode infection in rice: molecular and cellular insights. Annu. Rev. Phytopathol. 52, 7.1–7.19 (2014). 10.1146/annurev-phyto-102313-050111 24906129

[b6] Soriano I. R., Espiritu M. J., Schmidt V., Brar D. & Reversat G. Resistance to rice root-knot nematode *Meloidogyne graminicola* in *Oryza longistaminata* and *Oryza glaberrima*. Philippine J. Crop Sci. 23, 89 (1998).

[b7] Prasad J. S. *et al.* Root-knot nematode resistance in advanced back cross populations of rice developed for water stress conditions. Nematol. Mediterr. 34, 3–8 (2006).

[b8] Cabasan M. T. N., De Waele D. & Kumar A. Comparison of migration, penetration, development and reproduction of *Meloidogyne graminicola* on susceptible and resistant rice genotypes. Nematol. 14, 405–415 (2012).

[b9] Buchholz W. G., Teng W., Wallace D., Ambler J. & Hall T. C. In Methods in Molecular Biology: Plant Virology Protocols: from Virus Isolation to Transgenic Resistance, Vol. 81 (eds Foster G. & Taylor S.) Ch. 40, 383–396 (Humana Press Inc., 1998). 10.1385/0-89603-385-6:383 9760528

[b10] Rawat N., Himabindu K., Neeraja C. N., Nair S. & Bentur J. S. Suppressive subtraction hybridization reveals that rice gall midge attack elicits plant-pathogen-like responses in rice Plant Physiol. Biochem. 63, 122–130 (2013). 23257077 10.1016/j.plaphy.2012.11.021

[b11] Spence K. O., Lewis E. E. & Perry R. N. Host finding and invasion by entomopathogenic and plant-parasitic nematodes: evaluating the ability of laboratory bioassays to predict field results. J. Nematol. 40, 93–98 (2008). 19259525 PMC2586539

[b12] Wang C., Lower S. & Williamson V. M. Application of pluronic gel to the study of root-knot nematode behavior. Nematol. 11, 453–464 (2009).

[b13] Wang C., Lower S., Thomas V. P. & Williamson V. M. Root-knot nematodes exhibit strain-specific clumping behavior that is inherited as a simple genetic trait. PLoS ONE 5, e15148 (2010). 21151553 10.1371/journal.pone.0015148 PMC3000325

[b14] Dutta T. K., Powers S. J., Kerry B. R., Gaur H. S. & Curtis R. H. C. Comparison of host recognition, invasion, development and reproduction of *Meloidogyne graminicola* and *M. incognita* on rice and tomato. J. Nematol., 13, 509–520 (2011).

[b15] Reynolds A. M. *et al.* Chemotaxis can take plant-parasitic nematodes to the source of a chemo-attractant via the shortest possible routes. J. R. Soc. Interface 8, 568–577 (2011). 20880854 10.1098/rsif.2010.0417 PMC3061123

[b16] Ko M. P. & Van Gundy S. D. An alternative gelling agent for culture and studies of nematodes, bacteria, fungi, and plant tissues. J. Nematol. 20, 478–485 (1988). 19290241 PMC2618828

[b17] Goto D. B., Miyazawa H., Mar J. C. & Sato M. Not to be suppressed? Rethinking the host response at a root-parasite interface. Plant Sci. 213, 9–17 (2013). 24157203 10.1016/j.plantsci.2013.08.004

[b18] Goverse A. & Smant G. The activation and suppression of plant innate immunity by parasitic nematodes. Annu. Rev. Phytopathol. 52, 243–265 (2014). 24906126 10.1146/annurev-phyto-102313-050118

[b19] Li R. *et al.* Integrated signaling networks in plant responses to sedentary endoparasitic nematodes: a perspective. Plant Cell Rep. 34, 5–22 (2015). 25208657 10.1007/s00299-014-1676-6

[b20] Xiong L. & Yang Y. Disease resistance and abiotic stress tolerance in rice are inversely modulated by an abscisic acid-inducible Mitogen-Activated Protein Kinase. Plant Cell 15, 745–759 (2003). 12615946 10.1105/tpc.008714 PMC150027

[b21] Delteil A., Zhang J., Lessard P. & Morel J. B. Potential candidate genes for improving rice disease resistance. Rice 3, 56–71 (2010).

[b22] Liu W. *et al.* Recent progress in understanding PAMP- and effector-triggered immunity against the rice blast fungus *Magnaporthe oryzae*. Mol. Plant. 6, 605–620 (2013). 23340743 10.1093/mp/sst015

[b23] Lee H. I., Leon J. & Raskin I. Biosynthesis and metabolism of salicylic acid. Proc. Natl. Acad. Sci. USA 92, 4076–4079 (1995). 11607533 10.1073/pnas.92.10.4076 PMC41889

[b24] Wubben M. J., Jin J. & Baum T. J. Cyst nematode parasitism of *Arabidopsis thaliana* is inhibited by salicylic acid (SA) and elicits uncoupled SA-independent pathogenesis-related gene expression in roots. Mol. Plant-Microbe interact. 21, 424–432 (2008). 18321188 10.1094/MPMI-21-4-0424

[b25] Vlot A. C., Dempsey D. A. & Klessig D. F. Salicylic acid, a multifaceted hormone to combat disease. Annu. Rev. of Phytopathol. 47, 177–206 (2009). 19400653 10.1146/annurev.phyto.050908.135202

[b26] Dong X. N. NPR1, all things considered. Curr. Opin. Plant Biol. 7, 547–552 (2004). 15337097 10.1016/j.pbi.2004.07.005

[b27] Yuan Y. X. *et al.* Functional analysis of rice NPR1-like genes reveals that OsNPR1 ⁄ NH1 is the rice orthologue conferring disease resistance with enhanced herbivore susceptibility. Plant Biotechnol. J. 5, 313–324 (2007). 17309686 10.1111/j.1467-7652.2007.00243.x

[b28] Mei C. S., Qi M., Sheng G. Y. & Yang Y. N. Inducible overexpression of a rice allene oxide synthase gene increases the endogenous jasmonic acid level, PR gene expression, and host resistance to fungal infection. Mol. Plant-Microbe interact. 19, 1127–1137 (2006). 17022177 10.1094/MPMI-19-1127

[b29] Seo H. S. *et al.* Jasmonic acid carboxyl methyltransferase: a key enzyme for jasmonate-regulated plant responses. Proc. Natl. Acad. Sci. USA 98, 4788–4793 (2001). 11287667 10.1073/pnas.081557298 PMC31912

[b30] Lee M. W., Qi M. & Yang Y. O. A novel jasmonic acid-inducible rice myb gene associates with fungal infection and host cell death. Mol. Plant-Microbe interact, 14, 527–535 (2001). 11310740 10.1094/MPMI.2001.14.4.527

[b31] Iwai T., Miyasaka A., Seo S. & Ohashi Y. Contribution of ethylene biosynthesis for resistance to blast fungus infection in young rice plants. Plant Physiol. 142, 1202–1215 (2006). 17012402 10.1104/pp.106.085258 PMC1630725

[b32] Jun S. H. *et al.* OsEIN2 is a positive component in ethylene signaling in rice. Plant Cell Physiol. 45, 281–289 (2004). 15047876 10.1093/pcp/pch033

[b33] Hu Y. B., Zhao L. F., Chong K. & Wang T. Overexpression of OsERF1, a novel rice ERF gene, upregulates ethylene-responsive gene expression besides affects growth and development in Arabidopsis. J. Plant Physiol. 165, 1717–1725 (2008). 18313797 10.1016/j.jplph.2007.12.006

[b34] Mitsuhara I. *et al.* Characteristic expression of twelve rice PR1family genes in response to pathogen infection, wounding, and defence-related signal compounds (121/180). Mol. Genet. Genomics 279, 415–427 (2008). 18247056 10.1007/s00438-008-0322-9 PMC2270915

[b35] Shimono M. *et al.* Rice WRKY45 plays a crucial role in benzothiadiazole-inducible blast resistance. Plant Cell 19, 2064–2076 (2007). 17601827 10.1105/tpc.106.046250 PMC1955718

[b36] Qiu D. *et al.* Exploring transcriptional signalling mediated by OsWRKY13, a potential regulator of multiple physiological processes in rice. BMC Plant Biol. 9, 74 (2009). 19534828 10.1186/1471-2229-9-74 PMC3224702

[b37] Wuyts N., Lognay G., Swennen R. & De Waele D. Nematode infection and reproduction in transgenic and mutant Arabidopsis and tobacco with an altered phenylpropanoid metabolism. J. Exp. Bot. 57, 2825–2835 (2006). 16831845 10.1093/jxb/erl044

[b38] Wuyts N. *et al.* Potential physical and chemical barriers to infection by the burrowing nematode *Radopholus similis* in roots of susceptible and resistant banana (Musa spp.). Plant Pathol. 56, 878–890 (2007).

[b39] Portillo M. *et al.* Distinct and conserved transcriptomic changes during nematode-induced giant cell development in tomato compared with Arabidopsis: a functional role for gene repression. New Phytol. 197, 1276–1290 (2013). 23373862 10.1111/nph.12121

[b40] Hao P. *et al.* Herbivore-induced callose deposition on the sieve plates of rice: an important mechanism for host resistance. Plant Physiol. 146, 1810–1820 (2008). 18245456 10.1104/pp.107.111484 PMC2287352

[b41] Dixon R. A. & Paiva N. L. Stress-induced phenylpropanoid metabolism. Plant Cell 7, 1085–1097 (1995). 12242399 10.1105/tpc.7.7.1085 PMC160915

[b42] Sasaki-Crawley A. *et al.* The use of Pluronic F-127 to study the development of the potato cyst nematode, *Globodera pallida*. Nematol. 14, 869–873 (2012).

[b43] Khan M. R., Ashraf T. & Shahid S. Evaluation for relative susceptibility of rice against field population of *Meloidogyne graminicola. Indian* J. Nematol. 42, 46–52 (2012).

[b44] Nahar K., Kyndt T., De Vleesschauwer D., Hofte M. & Gheysen G. The jasmonate pathway is a key player in systemically induced defence against root-knot nematodes in rice. Plant Physiol. 157, 305–316 (2011). 21715672 10.1104/pp.111.177576 PMC3165880

[b45] Nahar K., Kyndt T., Nzogela Y. B. & Gheysen G. Abscisic acid interacts antagonistically with classical defence pathways in rice-migratory nematode interaction. New Phytol. 196, 901–913 (2012). 22985247 10.1111/j.1469-8137.2012.04310.x

[b46] Kyndt T. *et al.* Transcriptional reprogramming by root knot and migratory nematode infection in rice. New Phytol. 196, 887–900 (2012). 22985291 10.1111/j.1469-8137.2012.04311.x

[b47] Kyndt T. *et al.* Comparing systemic defence-related gene expression changes upon migratory and sedentary nematode attack in rice. Plant Biol. 14, 73–82 (2012). 22188265 10.1111/j.1438-8677.2011.00524.x

[b48] Nguyễn P. V. *et al.* *Meloidogyne incognita*-rice (*Oryza sativa*) interaction: a new model system to study plant–root-knot nematode interactions in monocotyledons. Rice 7, 23 (2014). 26224554 10.1186/s12284-014-0023-4 PMC4884005

[b49] Zhang S. & Klessig D. F. MAPK cascades in plant defence signalling. Trends Plant Sci. 6, 520–527 (2001). 11701380 10.1016/s1360-1385(01)02103-3

[b50] Bari R. & Jones J. D. Role of plant hormones in plant defence responses. Plant Mol. Biol. 69, 473–488 (2009). 19083153 10.1007/s11103-008-9435-0

[b51] Pieterse C. M. J., Leon-Reyes A., Van der Ent S. & Van Wees S. C. M. Networking by small-molecule hormones in plant immunity. Nat. Chem. Biol. 5, 308–316 (2009). 19377457 10.1038/nchembio.164

[b52] van Loon L. C., Rep M. & Pieterse C. M. J. Significance of inducible defence-related proteins in infected plants. Annu. Rev. Phytopathol. 44, 135–162 (2006). 16602946 10.1146/annurev.phyto.44.070505.143425

[b53] Tamogami S., Rakwal R. & Kodama O. Phytoalexin production elicited by exogenously applied jasmonic acid in rice leaves (*Oryza sativa* L.) is under the control of cytokinins and ascorbic acid. FEBS Lett. 412, 61–64 (1997). 9257690 10.1016/s0014-5793(97)00743-6

[b54] Schweizer P., Buchala A., Dudler R. & Metraux J. P. Induced systemic resistance in wounded rice plants. Plant J. 14, 475–481 (1998).

[b55] Lee A. *et al.* Inverse correlation between jasmonic acid and salicylic acid during early wound response in rice. Biochem. Biophys. Res. Commun. 318, 734–738 (2004). 15144900 10.1016/j.bbrc.2004.04.095

[b56] Sanz-Alferez S., Mateos B., Alvarado R. & Sanchez M. SAR induction in tomato plants is not effective against root-knot nematode infection. Euro. J. Plant Pathol. 120, 417–425 (2008).

[b57] Hamamouch N., Li C. Y., Seo P. J., Park C. M. & Davis E. L. Expression of Arabidopsis pathogenesis-related genes during nematode infection. Mol. Plant Pathol. 12, 355–364 (2011). 21453430 10.1111/j.1364-3703.2010.00675.x PMC6640486

[b58] Molinari S., Fanelli E. & Leonetti P. Expression of tomato salicylic acid (SA)-responsive pathogenesis-related genes in *Mi-1-*mediated and SA-induced resistance to root-knot nematodes. Mol. Plant Pathol. 15, 255–264 (2014). 24118790 10.1111/mpp.12085 PMC6638815

[b59] Gheysen G. & Jones J. In Plant nematology 1st edn (eds Perry R. N. & Moens M.) Ch. 9, 234–254 (CABI, 2006).

[b60] Bekal S., Niblack T. L. & Lambert K. N. A. A chorismate mutase from the soybean cyst nematode *Heterodera glycines* shows polymorphisms that correlate with virulence. Mol. Plant-Microbe Interact. 16, 439–446 (2003). 12744515 10.1094/MPMI.2003.16.5.439

[b61] Hewezi T. *et al.* Arabidopsis spermidine synthase is targeted by an effector protein of the cyst nematode *Heterodera schachtii*. Plant Physiol. 152, 968–984 (2010). 19965964 10.1104/pp.109.150557 PMC2815906

[b62] Jaouannet M. *et al.* The root-knot nematode calreticulin Mi-CRT is a key effector in plant defence suppression. Mol. Plant-Microbe Interact. 26, 97–105 (2013). 22857385 10.1094/MPMI-05-12-0130-R

[b63] Byrd D. W., Kirkpatrick T. & Barker K. R. An improved technique for clearing and staining plant tissues for detection of nematodes. J. Nematol. 15, 142–143 (1983). 19295781 PMC2618249

[b64] Livak K. J. & Schmittgen T. D. Analysis of relative gene expression data using real-time quantitative PCR and the 2(-Delta Delta C(T)) Method. Methods 25, 402–408 (2001). 11846609 10.1006/meth.2001.1262

