# Supplementary material for: Comparing the defence-related gene expression changes upon root-knot nematode attack in susceptible versus resistant cultivars of rice
Source: Sci Rep. 2016 Mar 10;6:22846. doi: 10.1038/srep22846 (PMC4785349; doi:10.1038/srep22846)
Supplement: Supplementary Information [file srep22846-s1.pdf]

SUPPLEMENTARY INFORMATION

Title: **Comparing the defence-related gene expression changes upon root-knot nematode attack in susceptible *versus* resistant cultivars of rice**

Authors:
Chanchal Kumari[1,2], Tushar K. Dutta[1], Prakash Banakar[1] and Uma Rao[1,*]

Affiliations:
[1]Division of Nematology, ICAR-Indian Agricultural Research Institute, New Delhi, India, 110012
[2]School of Biotechnology, Kalinga Institute of Industrial Technology, Bhubaneswar, India, 751024

**Corresponding Author*
Dr. Uma Rao
Division of Nematology
ICAR-Indian Agricultural Research Institute
New Delhi, India-110012
TEL: +91-112-584-2721
Email: umarao@iari.res.in; umanema@gmail.com

Supplementary Table S1. Primers used for gene expression studies.

Gene	Locus ID	Forward primer (5′-3′)	Reverse primer (5′-3′)
Os18srRNA	GenBank: AF069218	CGCGCAAATTACCCAATCCTGACA	TCCCGAAGGCCAACGTAAATAGGA
Os-actin	RAP-DB: Os03g0718100	CTCTCAGCACATTCCAGCAG	AGGAGGACGGCGATAACAG
OsMAPK5a	RAP-DB: Os03g17700	GTCTGCTCCGTGATGAAC	TGATGCCTATGATGTTCTCG
OsMAPK6	RAP-DB: Os06g0154500	GATACATTCGCCAACTTCC	CAGTGATGCCAGGTAAGG
OsMPAK20	RAP-DB: Os01t0629900	TCAACTCCAATTCCTGCCAAG	AACAACTCTTCCTGGTCTTGC
OsPAL1	RAP-DB: Os02g41630	TGTGCGTGCTTCTGCTGCTG	AGGGTGTTGATGCGCACGAG
OsICS1	RAP-DB: Os09g19734	TGTCCCCACAAAGGCATCCTGG	TGGCCCTCAACCTTTAAACATGCC
OsEDS1	RAP-DB: Os09t0392100	CAGGAGAGGCAGTGTTAATCG	GCAAGCGGAGTAAGTGGTATG
OsPAD4	RAP-DB: Os11t0195500	TCAGAGGCAAGGCAGTAGTG	ACCGCTCACGCAGGATAG
OsNPR1	RAP-DB: Os01t0194300	AGAAGTCATTGCCTCCAG	ACATCGTCAGAGTCAAGG
OsAos2	RAP-DB: Os03t0225900	GCGAGAGACGGAGAACCC	CGACGAGCAACAGCCTTC
OsJMT1	RAP-DB: Os06g0314600	CACGGTCAGTCCAAAGATGA	CTCAACCGTTTTGGCAAACT
OsJAMYB	RAP-DB: Os11g45740	GAGGACCAGAGTGCAAAAGC	CATGGCATCCTTGAACCTCT
OsACS1	RAP-DB: Os03g51740	GATGGTCTCGGATGATCACA	GTCGGGGGAAAACTGAAAAT
OsACO7	RAP-DB: Os01g39860	GGACTACTACCAGGGCACCA	GATTAGCGCACGCGATTTTA
OsEIN2	RAP-DB: Os07g06130	TAGGGGGACTTTGACCATTG	TGGAAGGGACCAGAAGTGTT
OsERF1	RAP-DB: Os04g46220	AAGGGTCATAATTCGCGTCA	TCCACACCACAAGACATCGT
OsPR1a	GenBank: EF061246	AACTTCGTCGGCCAATCTC	CATGCATAAACACGTAGCATAGC
OsPR1b	GenBank: EF061247	TACGACTACGCCTCCAACA	CCGGCTTATAGTTGCATGTGA
OsPR10	GenBank:AF274850	ACGCCTAAGATGAAGAGGAATAC	CTCAAACGCCACGAGAATTTG
OsWRKY13	RAP-DB: Os01t0750100	GCCAGCGGAGAACGAATC	CTCCTCCTGCTTCACAACC
OsWRKY45	RAP-DB: Os05g0322900	AATTCGGTGGTCGTCAAGAA	AAGTAGGCCTTTGGGTGCTT
OsC4H	RAP-DB: Os05g0320700	CAGACTGGTGAGATCCGGTG	TTCCCCATTCGATCGACCAC
OsCAD6	RAP-DB: Os04g0229100	TCGGTAAGAGGACGGTGAGT	TGTCGATGTCCCAGGTGATG
OsGSL1	GenBank: AP001389	TGAGGACCTGCCACGATT	CACGCTGATTGCGAACAT
OsGSL3	GenBank: AP003268	TGGCAAGCGACCACATAG	AGACCTTAGCACGGACTG
OsGSL5	GenBank: AP008212	GTGGTGTCCCTGCTATGA	GTTGTTTGCTATTCTCCC
OsGNS5	GenBank: U72251	TTGCGGCCATTCCTACAGT	TGGTGAGGGCGATGCTTG
